# Factors associated with sex work involvement among transgender women in Jamaica: a cross-sectional study

**DOI:** 10.7448/IAS.20.01/21422

**Published:** 2017-04-06

**Authors:** Carmen H Logie, Ying Wang, Ashley Lacombe-Duncan, Nicolette Jones, Uzma Ahmed, Kandasi Levermore, Ava Neil, Tyrone Ellis, Nicolette Bryan, Annecka Marshall, Peter A Newman

**Affiliations:** ^a^ Factor-Inwentash Faculty of Social Work, University of Toronto, Toronto, Canada; ^b^ Women’s College Research Institute, Women’s College Hospital, University of Toronto, Toronto, Canada; ^c^ Jamaica AIDS Support for Life, Kingston, Jamaica; ^d^ Women’s Empowerment for Change (WE-Change), Kingston, Jamaica; ^e^ Institute for Gender and Development Studies, University of the West Indies, Mona Campus, Kingston, Jamaica

**Keywords:** transgender, transgender women, HIV, sex work, transactional sex, structural drivers, Jamaica, violence

## Abstract

**Introduction**: Transgender women are disproportionately impacted by HIV. Transgender women involved in sex work may experience exacerbated violence, social exclusion, and HIV vulnerabilities, in comparison with non-sex work-involved transgender women. Scant research has investigated sex work among transgender women in the Caribbean, including Jamaica, where transgender women report pervasive violence. The study objective was to examine factors associated with sex work involvement among transgender women in Jamaica.

**Methods**: In 2015, we implemented a cross-sectional survey using modified peer-driven recruitment with transgender women in Kingston and Ocho Rios, Jamaica, in collaboration with a local community-based AIDS service organization. We conducted multivariable logistic regression analyses to identify factors associated with paid sex and transactional sex. Exchanging oral, anal or vaginal sex for money only was categorized as paid sex. Exchanging sex for survival needs (food, accommodation, transportation), drugs or alcohol, or for money along with survival needs and/or drugs/alcohol, was categorized as transactional sex.

**Results**: Among 137 transgender women (mean age: 24.0 [SD: 4.5]), two-thirds reported living in the Kingston area. Overall, 25.2% reported being HIV-positive. Approximately half (*n* = 71; 51.82%) reported any sex work involvement, this included sex in exchange for: money (*n* = 64; 47.06%); survival needs (*n* = 27; 19.85%); and drugs/alcohol (*n* = 6; 4.41%). In multivariable analyses, paid sex and transactional sex were both associated with: intrapersonal (depression), interpersonal (lower social support, forced sex, childhood sexual abuse, intimate partner violence, multiple partners/polyamory), and structural (transgender stigma, unemployment) factors. Participants reporting transactional sex also reported increased odds of incarceration perceived to be due to transgender identity, forced sex, homelessness, and lower resilience, in comparison with participants reporting no sex work involvement.

**Conclusions**: Findings reveal high HIV infection rates among transgender women in Jamaica. Sex work-involved participants experience social and structural drivers of HIV, including violence, stigma, and unemployment. Transgender women involved in transactional sex also experience high rates of incarceration, forced sex and homelessness in comparison with non-sex workers. Taken together, these findings suggest that social ecological factors elevate HIV exposure among sex work-involved transgender women in Jamaica. Findings can inform interventions to advance human rights and HIV prevention and care cascades with transgender women in Jamaica.

## Introduction

Globally, transgender women experience widespread stigma and discrimination that contributes to economic, social and health vulnerabilities [1]. Transgender women are defined as individuals who have been assigned male-at-birth and self-identify as female or as a transgender woman [2]. Transgender women experience social exclusion, stigma, and discrimination, and a lack of human rights protections that result in barriers to accessing adequate housing, education and employment [1,3,4]. Transphobia, in particular, fuels a lack of access to employment opportunities for transgender people, including job denial and on-the-job discrimination [5–7]. As a consequence of employment discrimination, transgender women may engage in sex work to acquire income, as well as use it as means of survival to acquire food, rent, shelter, drugs and alcohol [4,8,9]. Sex workers, broadly known as individuals who exchange sex for money or other goods, in general experience widespread rights violations and violence [10,11] due to the intersection of transgender stigma, sex work stigma and other marginalized identities [12,13].

Transgender women are disproportionately impacted by HIV; meta-analytic and systematic review results from transgender women (*n* = 7179) across ten low- and middle-income countries reported a pooled HIV prevalence of 17.7% (95% CI: 15.6–19.8), almost 49-fold higher than national estimated prevalence [14]. Figueroa’s study [15] with men who have sex with men (MSM) (*n* = 449) in Jamaica included 17 transgender participants with a reported HIV infection rate of 52.9%. Other systematic review findings suggest transgender women who are sex workers have significantly higher HIV infection rates in comparison with transgender women not involved in sex work; this review included one Caribbean country (Dominican Republic) [16]. HIV prevalence among transgender women sex workers is 9-fold higher than among non-transgender female sex workers [17]. There is limited information about transgender women’s sex work involvement in Jamaica, where homosexuality is criminalized and there are reports of pervasive stigma and violence targeting sexual and gender minorities, including transgender women [15,18]. The high reported HIV prevalence among transgender women in Jamaica, and stigmatizing social context, underscores the need for information on HIV vulnerabilities and prevention needs among transgender women involved in sex work in Jamaica.

Scholars have applied socio-ecological models to understand the complex, multi-directional interplay between individual, community- and structural-level factors, and their influence on health outcomes (e.g. HIV) and contexts of increased HIV exposure [19,20]. A recent review of HIV vulnerability among transgender sex workers globally employed a social ecological framework to understand contexts of HIV risk [12]. Structural factors (e.g. laws criminalizing sex work) indirectly influence and are influenced by interpersonal factors (e.g. community norms). At an interpersonal level, community norms may socially sanction violence against sex workers, which influences individual-level factors (e.g. mental health challenges, low self-efficacy). These structural, interpersonal, and individual factors converge with biological factors (e.g. receptive anal sex, condomless anal sex) to elevate HIV exposure [12]. A systematic review examining structural factors that elevate HIV exposure among female sex workers in Africa, Asia, Eastern Europe and Russia reported that criminalization of sex work and punitive policies limit where sex workers can work, and a lack of legal recognition contributes to economic and food insecurity as well as constraints in accessing material resources needed to maintain optimal health [21].

At the interpersonal level, a lack of legal protections as well as stigma have been associated with reduced condom use. Transgender women have less ability to negotiate condom use due to poverty and need for income from sex work, which increases their exposure to HIV infection [21]. A study with transgender women in three Sub-Saharan African countries reported that social stigma was associated with condomless anal sex and sex work, and stigma from family and friends was also associated with sex work – highlighting how structural (*social stigma and marginalization*) and interpersonal (*family and friend stigma*) factors may contribute to engagement in sex work and biological risks (*condomless anal sex*) [2]. Shannon [21] also discusses how punitive policies and police violence may lead to raids that result in confiscation of condoms or clean needles, limiting access to HIV prevention tools.

At the intrapersonal level, pervasive discrimination and stigma may be internalized and impact mental wellbeing among transgender sex workers. Stigma, social exclusion and violence can contribute to depression, anxiety, substance use and high rates of suicidal ideation, eating disorders and post-traumatic stress disorder [1,12,22]. Substance use may be a coping mechanism in the face of widespread discrimination, further amplifying negative health outcomes [1]. A US study with transgender and transsexual participants with a history of sex work reported that depression was associated with transphobia and lower levels of social support, suggesting that social support is a protective factor that can promote mental wellbeing [23]. Studies conducted within Canada and the US also suggest that transgender people use adaptive coping strategies to manage, and reduce the negative impacts of, stigma, including resiliency (e.g. enhancing one’s inner ability to bounce back from negative life situations) and social support (e.g. connecting and building relationships and solidarity with others) [6,13,24].

Structural, interpersonal and intrapersonal factors therefore operate separately as well as interact to elevate HIV risk among transgender women [14,25,26] and female sex workers, including transgender women sex workers [10,12,26]. Sex workers and transgender women are key populations to engage in the HIV care cascade; the overrepresentation of these populations in the global HIV pandemic is produced by social and structural inequities [1,12,25–27]. Scant research across the globe has collected information on sex work among transgender women, precluding understanding HIV vulnerabilities and HIV prevention needs among this population [12,26]. There are knowledge gaps regarding sex work engagement among transgender women in the Caribbean, in particular Jamaica. Our study objective was to understand intrapersonal, interpersonal and structural factors associated with sex work involvement among transgender women in Jamaica.

## Methods

### Setting and study design

This cross-sectional study was implemented in collaboration with Jamaica AIDS Support for Life, a community-based national AIDS service organization in Jamaica, from March 2015 to October 2015 in Kingston, Ocho Rios and surrounding areas. Kingston is the capital and largest city of Jamaica, home to one-fifth of Jamaica’s population at approximately 600,000 inhabitants [28]. Ocho Rios has a small population of under 10,000 people [29], but has a large influx of tourists due to tourist resorts. Surrounding areas to Kingston are characteristically more urban, whereas surrounding areas to Ocho Rios are more rural. Thus, this sample of transgender women was recruited from a mix of settings [30]. No studies to-date have documented the experiences of transgender sex workers in Jamaica. However, community-based organizations have documented how stigma, criminalization and police brutality shape the HIV vulnerability of Jamaican sex workers [31].

We hired and trained 7 peer research assistants (PRAs) who self-identified as a sexual and/or gender minority (lesbian, gay, bisexual, transgender; LGBT) person from Kingston, Ocho Rios and surrounding areas. PRA were trained in research ethics and methods, and assisted with development and selection of survey measures to enhance appropriateness and clarity for the local context and transgender population, participant recruitment and survey implementation. PRAs received training over 4 days, with regards to conducting in-depth interviews (16 h) and implementing computerized (tablet-based) data collection methods (16 h). PRAs were provided with weekly supervision from the research coordinators in Kingston and Ocho Rios. Data quality was assessed at three time points during data collection, after which PRAs received further in-person training in booster sessions (two 8 h sessions, bi-monthly) as well as weekly supervision to discuss data quality issues and data collection procedures.

We used purposive, non-random sampling methods to access this marginalized population [32]. PRAs used snowball sampling to recruit transgender participants – participants self-identified as labelled male-at-birth persons now identifying as women – from their social networks [33]. In addition to being members of the LGBT community, PRAs were HIV outreach workers at Jamaica AIDS Support for Life and provided mobile testing services with rapid HIV testing and HIV prevention materials (e.g. condoms, lubricant). PRAs shared study information with potential participants in their capacity as Jamaica AIDS Support for Life outreach workers. No print materials (i.e. posters, flyers) were used due to the lack of legal protections and rights for transgender persons in Jamaica.

To supplement PRA recruitment with this hidden population, we included modified peer driven recruitment sampling methods [34]. Participants were notified they could invite a maximum of five other participants to the study with study coupons; participants received $500 Jamaican dollars (approximately $4 USD) per recruited participant. We aimed to recruit 150 participants; using G*Power software [35] a sample size of 148 was calculated as sufficient for logistic regression (OR 2.0, *p* < 0.05, power 0.95).

PRAs administered structured 30-minute interviews on android tablets using FluidSurveys^TM^ in a location of the participant’s choosing (e.g. home, park, Jamaica AIDS Support for Life). Participants received $1000 Jamaican dollars (approximately $8 USD) to complete the survey. PRA read the informed consent information aloud to the participant from the tablet; before being permitted to complete the survey, eligible participants provided voluntary written informed consent on the tablet. Research ethics approval was provided from the University of Toronto, Toronto, Canada and the University of the West Indies, Mona Campus, Kingston, Jamaica.

### Participants and eligibility

Eligibility criteria included persons who identified as a transgender woman (biologically assigned male sex at birth and self-identify as a woman), residing in Jamaica, 18 years and over, and able to provide informed consent.

### Survey measures

To assess sex work involvement, we used the question: “In the last year have you ever had sex (oral, vaginal, or anal) in exchange for (please check all that apply): (a) a place to stay, food or transportation (survival needs); (b) money; (c) drugs or alcohol; (d) none of the above”. Participants that reported “none of the above” were coded as not involved in sex work (0). Participants that only reported only (b), “money”, were coded as involved in paid sex (1). Participants that reported sex work for (a) survival needs, and/or (c) drugs/alcohol, or for (b) money along with (a) survival needs and/or (c) drugs/alcohol, were coded as involved in transactional sex (2). These three categories were pairwise mutually exclusive and were collectively exhaustive.

We assessed socio-demographic variables, including age and monthly income (measured continuously) in Jamaican dollars (converted to US dollars for analysis), city of residence (Kingston, Ochos Rios, with the option to list other places of residence), and highest level of education.

We examined intrapersonal factors, including screening for depression symptoms over the past 2 weeks using the two-item Patient Health Questionnaire-2 (PHQ-2) [36] (Cronbach’s α = 0.67; scale range 0–8). HIV and STI testing history was assessed by asking if persons had received an HIV test, or STI test not including HIV, in their lifetime. Participants self-reported their HIV status and a lifetime history of STI. Resilience was assessed by using The Brief Resilience Scale [37] to assess the ability to bounce back after stress (Cronbach’s α = 0.77; scale range 6–30).

We assessed interpersonal factors, including relationship status, consistency of condom use during anal sex with men over the past 3 months (usually versus usually not, with usually classified as consistent), number of lifetime sex partners, experiences of childhood sexual abuse, physical violence in adulthood, and forced sex (“In your life have you ever experienced forced sex, for example rape, sexual assault?”). We used a brief social support sub-scale to assess unmet social support needs; this scale was previously assessed for validity and reliability in a Caribbean context (Puerto Rico) [38] (Cronbach’s α = 0.83; range 5–35).

To assess structural factors such as transgender stigma, we adapted Diaz et al.’s [39] Homophobia Scale to measure enacted (Cronbach’s α = 0.77; range 7–28) and perceived stigma (Cronbach’s α = 0.61; range 5–20) towards transgender persons. The adaptation involved replacing each item that attributed stigma “because of your homosexuality” to “because you are transgender”. Enacted stigma refers to discriminatory acts, including violence and mistreatment. The perceived transgender stigma scale assesses awareness of negative norms, attitudes, beliefs and treatment towards transgender persons, and includes items such as: “How often have you heard that transgender people are not normal?” The enacted transgender stigma scale assesses acts of discrimination, violence and mistreatment, and includes items such as: “How often have you been hit or beaten up for being transgender?” We conducted confirmatory factor analysis (CFA) to assess the transgender scale factor structure, and results indicate a good model fit (TLI = 0.94, CFI = 0.88, RMSEA = 0.11). We asked for self-reported history of incarceration perceived to be due to transgender identity. Housing security was measured by asking: “In the past month, where have you usually slept? (a) In my own house or apartment; (b) in other people’s house or apartment; (c) outside (homeless)”. We also assessed employment status.

### Statistical analysis

We conducted descriptive analyses for socio-demographic and outcome variables in order to determine frequencies and proportions, and means and standard deviations, for participants with available data. We conducted bivariate analysis (T-test, chi square) to compare the difference between participants who never engaged in any sex work to those who had engaged in sex work. We conducted multinomial logistic regression to determine the odds ratio for sex work involvement among transgender women in Jamaica, controlling for socio-demographic characteristics (income, employment status, relationship status). We report the adjusted odds ratios, 95% confidence intervals, and *p*-values. The base outcome/reference group selected for the regression models were participants with no sex work involvement. Missing responses were excluded from the analyses. All statistical analyses were performed using STATA (version12.0) [40].

## Results

### Study population

The mean age of the 137 study participants was 24.0 years (SD: 4.5; range 18–44). Over half (*n* = 71; 51.8%) of participants reported any sex work involvement, of which, 42 (59.2%) were involved in sex only in exchange for money (categorized as paid sex) and 29 (40.1%) were involved in transactional sex. Two-thirds of the sample (67.2%) lived in the Kingston area. Participants reported an average monthly income of $225.5 USD (SD: 337.9). Table 1 reports participant socio-demographic characteristics by those reporting any sex work involvement (*n* = 71) and those with no sex work involvement (*n* = 66). In comparison with participants with no sex work involvement, sex work-involved participants reported: lower income and education; higher unemployment, homelessness, and incarceration perceived to be due to transgender identity; and were more likely to report having multiple partners or being in a polyamorous relationship.

**Table 1.** Main characteristics of participants by sex work involvement (*n* = 137).

		**No sex work involvement** (*n* = 66)	**Any sex work involvement** (*n* = 71)	
Variables		Mean (SD)^a^	Mean (SD) ^a^	*p*-value (T-test, Chi-square)
Age (*n* = 130)		Mean = 24.3 (SD = 4.3), *n* = 63	Mean = 24.2 (SD = 4.8), *n* = 67	0.985
Monthly income in US Dollars (*n* = 114)		Mean = 310.9 (SD = 419.2) *n* = 56	Mean = 128.2 (SD = 161.4) *n* = 58	0.003
		Frequency (%)^a^	Frequency (%)^a^	
City of residence				0.018
	Kingston	47 (71.2)	45 (63.4)	
	Montego Bay	1 (1.5)	2 (2.8)	
	Ochos Rios	7 (10.6)	20 (28.2)	
	Spanish Town	6 (9.1)	4 (5.6)	
	Others	5 (7.6)	0	
Education (*n* = 135)		*n* = 65	*n* = 70	0.001
	Less than high school	5 (7.7)	21 (30.0)	
	Completed high school	31 (47.7)	36 (51.4)	
	Some college	12 (18.5)	8 (11.4)	
	Completed college or higher	17 (26.2)	5 (7.1)	
Employment (*n* = 130)		*n* = 62	*n* = 68	<0.001
	Employed full time	27 (43.6)	9 (13.2)	
	Employed part time	11 (17.7)	25 (36.8)	
	Student	10 (16.1)	4 (5.9)	
	Unemployed	14 (22.6)	30 (44.1)	
Place of residence (*n* = 134)		*n* = 65	*n* = 69	0.001
	In my own house or apartment	22 (33.9)	8 (11.6)	
	In other people’s homes or apartment	41 (63.1)	49 (71.0)	
	Outside (homeless)	2 (3.1)	12 (17.4)	
Relationship status (*n* = 136)		*n* = 65	*n* = 71	0.008
	Married or living together	16 (24.6)	11 (15.5)	
	Dating-not living together	21 (32.3)	17 (23.9)	
	Casual dating	7 (10.8)	7 (9.9)	
	No current partner	17 (26.2)	14 (19.7)	
	Multiple partners/polyamorous	4 (6.2)	22 (31.0)	
Ever incarcerated perceived to be due to transgender identity		8 (12.1)	23 (32.4)	0.005
Sexual orientation		*n* = 63	*n* = 67	0.130
	Heterosexual	21 (33.3)	19 (28.4)	
	Bisexual	11 (17.5)	4 (6.0)	
	Lesbian/Gay	25 (39.7)	37 (55.2)	
	Queer	6 (9.5)	7 (10.5)	
Sex work involvement		*n* = 66	*n* = 71	
	Not involved in sex work	66 (100)	0	–
	Paid sex		42 (59.2)	
	Transactional sex work		29 (40.8)	

^a^Percentages calculated from non-missing responses for each variable.

Table 2 illustrates HIV vulnerability and HIV and STI prevalence and testing among the sample. Overall, 25.2% (*n* = 26) of persons ever having received an HIV test (*n* = 103) self-reported being HIV positive, and 13.3% (*n* = 11) of persons who reported having received a test for sexually transmitted infections (not including HIV) (*n* = 83) self-reported a lifetime STI history. There were no differences in HIV or STI testing or outcomes between sex work-involved and non-sex work-involved participants, and no differences in condom use. Sex work-involved participants reported higher forced sex and greater number of sex partners than participants not involved in sex work.

**Table 2.** HIV and STI vulnerabilities and testing history by sex work involvement (*n* = 137).

Variables	**No sex work involvement** (*n* = 66) Frequency (%)	**Sex work involvement** (*n* = 71) Frequency (%)	*p*-Value
HIV positive serostatus (*n* = 103)	10 (20.4), *n* = 49	16 (29.6), *n* = 54	0.282
Lifetime history of STI (*n* = 83)	5 (12.8), *n* = 39	6 (13.6), *n* = 44	0.913
Consistent condom use during anal sex with men in past three months	42 (91.3)	57 (93.4)	0.677
Number of lifetime sexual partners (*n* = 136)	Mean = 11.4 (SD = 14.8), *n* = 66	Mean = 26.9 (SD = 32.2), *n* = 70	<0.001
Ever experienced forced sex	18 (27.3)	46 (64.8)	<0.001
Received HIV test ever	49 (74.2)	54 (76.1)	0.806
Received STI test ever	39 (59.1)	44 (62.0)	0.730

### Multinomial logistic regression modelling of sex work involvement among transgender women in Jamaica

Unadjusted and adjusted multinomial logistic regression results are displayed in Table 3. The model is significant (*p* < 0.001). Univariate analysis indicated that sex work involvement was associated with monthly income, relationship status (multiple partners/polyamory vs. Married) and employment status (No source of income vs. Employed full time). Compared to participants with no sex work involvement, in multivariate analyses the odds ratios of both involvement in paid sex and transactional sex (for accommodation, food, transportation, drugs or alcohol, or money in addition to these factors) were associated with *intrapersonal* (depression), *interpersonal* (unmet social support needs, forced sex, childhood sexual abuse, interpersonal violence), and *structural* (enacted and perceived transgender stigma) factors.

**Table 3.** Multinomial logistic regression modelling for sex work involvement among transgender women (*n* = 137).

	Paid sex (*n* = 42)	Transactional sex (*n* = 29)	Paid sex (*n* = 42)	Transactional sex (*n* = 29)
Variables	Unadjusted OR (95% CI) (*n* = 42)	Unadjusted OR, (95% CI) (*n* = 29)	Adjusted OR^a^ (95% CI) (*n* = 42)	Adjusted OR,^a^ (95% CI) (*n* = 29)
**Socio-demographic factors**				
Age	1.0 (0.9–1.1)	0.9 (0.8–1.0)		
Monthly income	0.9 (0.9–0.9)*	0.9 (0.9–0.9)*		
Employment status				
Employed full time	1	1		
Employed part time	1.8 (3.3–35.8)***	1.8 (0.4–9.6)		
Student	0	2.7 (0.6–12.9)		
Received social assistance	10.8 (1.5–75.7)*	10.1 (1.3–80.6)*		
No source of income	5.0 (1.4–17.4)*	6.8 (1.8–25.3)**		
Relationship status				
Married or living together (ref)	1	1		
Dating-not living together	1.2 (0.4–3.7)	1.0 (0.2–4.2)		
Casual dating	1.1 (0.3–5.1)	2.3 (0.4–14.3)		
No current partner	0.7 (0.2–2.5)	2.5 (0.6–11.2)		
Multiple partners/polyamorous	5.5 (1.3–22.9)*	14.7 (2.7–78.9)**		
**Intrapersonal factors**				
Depression	1.7 (1.3–2.4)***	2.2 (1.6–3.1)***	1.7 (1.1–2.6)*	2.2 (1.5–3.3)***
Resilience	0.9 (0.8–0.9)***	0.8 (0.7–0.9)***	0.9 (0.8–1.0)	0.8 (0.7–0.9)*
**Interpersonal factors**				
Need for social support	1.1 (1.0–1.2)**	1.2 (1.1–1.3)***	1.1 (1.0–1.2)*	1.2 (1.1–1.4)**
Experienced forced sex	4.3 (1.9–9.9)***	5.9 (2.3–15.4)***	6.9 (2.3–20.6)**	13.7 (3.6–52.2)***
History of childhood sexual abuse	5.7 (2.1–15.5)**	3.8 (1.2–11.5)*	3.6 (1.3–9.5)*	3.9 (1.1–13.3)*
Experienced intimate partner violence	3.4 (1.5–7.6)**	3.8 (1.5–9.4)**	3.0 (1.1–8.1)*	4.2 (1.3–13.1)*
**Structural factors**				
Place of residence				
Own house or apartment (ref)	1	1	1	1
Other people’s house or apartment	3.8 (1.3–11.0)*	2.5 (0.7–9.7)	4.7 (1.5–14.34)**	1.5 (0.4–6.6)
Live outside (homeless)	4.4 (0.5–39.2)	36.7 (5.3–254.9)***	4.8 (0.5–43.5)	26.33 (3.6–191.0)
Enacted transgender stigma	1.2 (1.1–1.3)**	1.3 (1.1–1.4)***	1.2 (1.1–1.4)**	1.3 (1.1–1.5)***
Perceived transgender stigma	1.5 (1.3–1.7)***	1.3 (1.1–1.5)**	1.7 (1.4–2.2)***	1.3 (1.1–1.7)*
Ever incarcerated perceived to be due to transgender identity	2.3 (0.8–6.3)	5.9 (2.1–16.7)**	1.2 (0.3–4.0)	5.3 (1.3–21.0)*

^a^Controlled for monthly income, employment status and relationship status. *p < 0.05. **p < 0.01. ***p < 0.001.

Participants reporting transactional sex, in comparison with paid sex, had increased odds of depression symptoms. As illustrated in Figure S1, with the maximum score on the depression scale of 8, the probability of paid sex was 0.25 and the probability of engaging in transactional sex was 0.60. Transactional sex involvement, but not paid sex, was associated with lower resilience scores.

At the interpersonal level, participants involved in paid sex were nearly 7 times more likely to report forced sex than participants not involved in sex work. This odds ratio nearly doubled for persons involved in transactional sex, who were almost 14 times more likely than participants not involved in sex work to report forced sex.

At the structural level, participants involved in paid sex or transactional sex were more likely to report transgender stigma than participants not involved in sex work, and these odds of experiencing transgender stigma were higher with transactional sex. As shown in Figure S2, with the maximum enacted transgender stigma score of 28, the probability of engaging in paid sex was 0.43 and the figure of transactional sex involvement was 0.49.

Two structural factors were only significantly different between participants engaged in transactional sex and participants not involved in sex work: being incarcerated perceived to be due to transgender identity and living outside/being homeless. Participants who were involved in transactional sex were over 5 times more likely to report being incarcerated than non-sex work-involved participants. Participants involved in transactional sex had much greater odds of reporting living outside/homelessness than participants not involved in sex work.

## Discussion

This study is among the first to examine factors associated with sex work involvement among transgender women in Jamaica. We found one-quarter of participants were HIV positive and over half of participants were involved in sex work. This study identified social ecological [19] factors spanning intrapersonal, interpersonal and structural dimensions that are associated with sex work involvement among transgender women in Jamaica. Transgender women involved in sex work in Jamaica experience elevated exposure to social and structural drivers of HIV, including forced sex, incarceration perceived to be due to transgender identity, intimate partner violence, and homelessness – with greater risks among transgender women exchanging sex for survival needs, drugs or alcohol.

Our finding that over 47% of transgender participants reported sex work for money in the past year is similar to studies with transgender women that report rates of sex work from 40 to 60% in Peru [3] and 33% in El Salvador [41]. We found approximately one-quarter of participants reported transactional sex in exchange for food, transportation, accommodation, drugs or alcohol. This is similar to findings that approximately one-third of samples of transgender women in San Salvador, El Salvador (*n* = 670) [41], and MSM in Jamaica (*n* = 449) reported transactional sex [15].

Corroborating prior research, we found a range of intrapersonal, interpersonal and structural disparities with transgender women involved in sex work. Being involved in any kind of sex work was associated with depression, lower social support, forced sex, physical violence, childhood sexual abuse (CSA), and transgender stigma. Similar to other studies [23,42], we found increased depression and lower social support among sex work-involved transgender women. Specifically, we found that expressing a higher need for social support was associated with increased odds of paid sex and transactional sex, and participants engaged in transactional sex had the greatest need for social support. We found two-thirds of participants involved in any kind of sex work reported ever experiencing forced sex, consistent with prior literature among transgender women in Peru [2] and San Francisco [3,43]. This is higher than rates in Shannon’s [10] Canadian study that reported 49% of female sex workers experienced rape or sexual assault by clients in the past 18 months; this could be because we did not have the same timeframe or perpetrator restriction, or because of higher rates of poverty and survival sex, which may place persons engaging in sex work at even higher risk of violence, with less discretion to refuse sex or try to escape. Our forced sex finding is also higher than rates of 18.9% reported among MSM sex workers in Jamaica [15]. These differences suggest transgender women sex workers may be particularly vulnerable to sexual violence in Jamaica. These findings align with the documented co-occurrence of depression, a greater need for social support, and a history of childhood sexual abuse among transgender sex workers in the US [23]. These results call for a multi-faceted approach to interventions to: address mental health (depression) and experiences and impacts of violence (childhood, adulthood); reduce stigma; and build social support networks to promote wellbeing among transgender women sex workers in Jamaica.

Our finding of higher rates of sex work among participants with experiences of intimate partner violence corroborates prior research; a study with sex workers in South Africa reported higher rates of sex work among women with a history of intimate partner violence [44]. Our findings of higher CSA among sex workers corroborate prior literature that suggests CSA enhances vulnerability to homelessness, exploitation and survival sex work [45–47]. We found higher transgender stigma among sex work-involved participants, illustrated in Figure S2; this corroborates research that highlights how transphobia and gender identity discrimination by family members was significantly associated with engaging in sex work for money or drugs [4]. Higher transgender stigma among sex work-involved participants also suggests the importance of understanding and assessing intersectional stigma, particularly the convergence of transgender stigma and sex work stigma. Taken together, our findings point to high rates of violence and stigma across the life course among Jamaican transgender women.

We found transgender women in transactional sex work experienced greater homelessness – sleeping outside – and incarceration perceived to be due to transgender identity than those who exchanged sex for money only, and non-sex work-involved participants. These findings corroborate results from a cross-sectional study in Los Angeles and Chicago with 151 transgender female youth that reported homelessness and incarceration were independently associated with transactional sex [13,48], and research from Los Angeles with MSM that demonstrates associations between homelessness and sex work. Transactional sex has been associated with poor housing conditions among female sex workers in South Africa [44], and with homelessness among youth in Canada [45]. Higher incarceration perceived to be due to transgender identity, as well as higher depression, forced sex, and transgender stigma demonstrate increased social and health disparities among transgender women involved in transactional sex.

Similar to studies that identify resilience among transgender populations [6,24], we found that resilience may be a protective factor for sex work involvement among transgender women in Jamaica. Specifically, for each point increase in resilient coping, participants experienced 16% reduction in the odds of being engaged in transactional sex. This finding highlights the need for intrapersonal-level interventions focused on resilience for transgender women, to build confidence, self-esteem and coping skills that can provide additional livelihood opportunities.

We found no differences in condom use or HIV/STI testing between sex work-involved and non-sex work-involved participants, perhaps due to ongoing efforts in Jamaica to address HIV testing disparities among sex workers [31]. Thus, future studies may explore the experience of transgender stigma in HIV services as a potential barrier to access-to-care, as comprehensive sexual health services are important for sex workers. We did, however, find higher number of lifetime sex partners among sex work-involved participants, and sex work-involved participants were more likely to report having multiple partners or being in polyamorous relationships than non-sex work-involved participants. This suggests an area for intervention, as sex work-involved participants may need to use condoms more consistently and access HIV/STI testing more frequently due to increased risk of HIV and STI exposure.

There are study limitations to consider in interpreting the results. The cross-sectional study design limits understanding causality and directionality of factors associated with sex work. To access this marginalized and hidden population, we utilized non-random sampling; this limits the ability to generalize findings across transgender women in Jamaica. Participants self-selected into the study based on self-identified transgender woman identity, however, we did not ask questions about transgender identity such as whether participants identified as women sometimes or always, and how women express or their gender on a day-to-day basis. These additional factors may have enhanced our understanding of transgender women’s experience of gender in context. Some measures, such as condom use and HIV status, may be subject to social desirability bias whereby participants may have over-reported past three-month condom use during anal intercourse and under reported HIV status. A scaled measure of condom use such as one validated among a large sample (*n* = 1383) of commercial sex workers in the Philippines, may have mitigated social desirability bias [49]. Inclusion of point-of-care HIV testing may have mitigated the effects of social desirability bias, allowing for a more thorough understanding of the association between these factors and sex work among transgender women in Jamaica. Moreover, as condom use may vary by partner type among trans-women [50] and sex workers [51], future studies should also collect data with regards to transgender women’s partner type to inform nuanced interventions. Beyond HIV and STI testing, we did not measure access to care (such as primary care) among the full sample nor access to HIV-related care (such as anti-retroviral treatment) among transgender sex workers living with HIV. Future research in this area is necessary in light of attrition across the HIV care continuum for all Jamaicans whereby substantially less people living with HIV who are linked to care are retained in care and currently use anti-retroviral treatment [52]. Additionally, the fact that most participants who were sex work involved engaged in multiple types of sex work – both for money and for food, accommodation, sex or drugs – resulted in overlap between the sex work-involved categories; despite this overlap, however, we identified statistically significant differences between transgender women engaged in paid sex and those engaged in transactional sex. Finally, although we found higher rates of forced sex, and higher rates of HIV infection, among sex work-involved participants, HIV infection was not significantly associated with sex work involvement. The sample size was not sufficiently powered to detect HIV infection differences based on sex work involvement in this study.

Despite these limitations, this study is unique in investigating factors associated with different types of sex work among transgender women in Jamaica. Few studies of sex work discern between different types of sex work [53], precluding understanding of shared and different HIV vulnerabilities for transgender women involved in paid sex and transactional sex. Our findings highlight the urgent need to address HIV prevention, care and treatment, human rights, and survival needs among transgender women, and particularly transgender women sex workers, in Jamaica.

## Conclusions

Our study findings can inform research and practice with transgender women involved in sex work in Jamaica. Interventions can address structural drivers of HIV risk among transgender women sex workers in Jamaica – including reducing violence, stigma and incarceration – to promote wellbeing and reduce exposure to HIV. Addressing human rights of transgender women, particularly regarding reducing incarceration, forced sex and other forms of violence, and increasing access to economic and housing opportunities, can help to reduce HIV risk and provide safety and dignity [1,12,54,55]. Poteat et al.’s [12] review of HIV prevention interventions with transgender women sex workers highlights the importance of multilevel approaches to address structural and individual vulnerabilities, and calls for more research on behavioural HIV prevention interventions with transgender sex workers outside of North America. Our findings also corroborate prior research [12,27] that identified the need for community empowerment, social support, and affirmative health services to optimize HIV prevention, treatment and care for transgender sex workers. Future research with larger sample sizes and point-of-care HIV and STI testing could more accurately ascertain HIV and STI prevalence, and assess their association with sex work.

Future research with local Jamaica transgender communities, community groups, and AIDS service organizations can adapt regional blueprints developed for the Caribbean [27] to promote gender affirmation in trans-competent care and other health and social justice interventions for transgender women in the Jamaica context. Given the extreme vulnerability and marginalization of transgender sex workers in Jamaica, approaches should include increasing access to gender affirming health and social services and meaningfully engaging transgender communities in research and service delivery. Studies that explore the perceived quality of existing health and social services may also help to inform interventions to reduce stigma and promote access to care for transgender women. Initiatives to reduce stigma and violence, and promote social justice, should address comprehensive health outcomes (sexual, mental, emotional) as well as build strengths, empowerment and community among transgender women in Jamaica [1,12,27,54,55].

## Supplementary material

To access the supplementary material to this article please see Supplementary Files under Article Tools online.
